# Single Trial Predictors for Gating Motor-Imagery Brain-Computer Interfaces Based on Sensorimotor Rhythm and Visual Evoked Potentials

**DOI:** 10.3389/fnins.2016.00164

**Published:** 2016-04-26

**Authors:** Andrew Geronimo, Mst Kamrunnahar, Steven J. Schiff

**Affiliations:** ^1^Department of Engineering Science and Mechanics, Center for Neural Engineering, The Pennsylvania State UniversityUniversity Park, PA, USA; ^2^Department of Neurosurgery, Penn State College of MedicineHershey, PA, USA; ^3^Department of Physics, The Pennsylvania State UniversityUniversity Park, PA, USA

**Keywords:** brain-computer interface, electroencephalography, motor-imagery, visual evoked potential

## Abstract

For brain-computer interfaces (BCIs) that utilize visual cues to direct the user, the neural signals extracted by the computer are representative of ongoing processes, visual evoked responses, and voluntary modulation. We proposed to use three brain signatures for predicting success on a single trial of a BCI task. The first two features, the amplitude and phase of the pre-trial mu amplitude, were chosen as a correlate for cortical excitability. The remaining feature, related to the visually evoked response to the cue, served as a possible measure of fixation and attention to the task. Of these three features, mu rhythm amplitude over the central electrodes at the time of cue presentation and to a lesser extent the single trial visual evoked response were correlated with the success on the subsequent imagery task. Despite the potential for gating trials using these features, an offline gating simulation was limited in its ability to produce an increase in device throughput. This discrepancy highlights a distinction between the identification of predictive features, and the use of this knowledge in an online BCI. Using such a system, we cannot assume that the user will respond similarly when faced with a scenario where feedback is altered by trials that are gated on a regular basis. The results of this study suggest the possibility of using individualized, pre-task neural signatures for personalized, and asynchronous (self-paced) BCI applications, although these effects need to be quantified in a real-time adaptive scenario in a future study.

## 1. Introduction

The goal of a brain-computer interface (BCI) is to interpret the intention of a subject directly from neuronally generated signals, in order to develop a direct communication pathway between the brain and the external environment. Successful BCI implementation has enormous potential for improving the quality of life of certain disabled groups, including those with spinal cord injury and locked-in syndrome induced by stroke or late-stage amyotrophic lateral sclerosis (Birbaumer, 2006).

Motor-imagery is a specific type of mental rehearsal applied for BCI use, in which the intention of the subject is determined by their imagination of movement of a specific part of the body e.g., feet, arms, or tongue. Imagination of movement in these limbs results in changes in the sensorimotor rhythms (SMR), which are recorded using electroencephalography (EEG) from motor and premotor cortical areas. SMRs are commonly defined in two frequency bands, mu and beta. The actual frequencies are subject specific, but typically occur around 8–12 and 18–25 Hz, respectively (Pfurtscheller and da Silva, 1999; McFarland et al., 2000). The modulation of these rhythms in response to overt as well as imagined movement has been well documented (McFarland et al., 2000; Miller et al., 2010), and their utility in controlling BCIs of substantial complexity has been demonstrated (McFarland et al., 2010).

Success in controlling an SMR-based BCI system depends on the user's ability modulate these motor rhythms from a baseline state. The reasons for why some individuals are able to do this well and why others are not is an area of active research, with differences in imagery ability, baseline oscillations, brain structure, and motivation being implicated as possible factors (Halder et al., 2013). One such predictor of BCI performance is the level of baseline SMR amplitude, which has been shown to be positively correlated with the success of the individual in completing the task (Blankertz et al., 2010). Within individuals, high SMR amplitude has further been shown to be correlated with more successful single trial performance (Maeder et al., 2012).

EEG oscillations such as the SMR have been given roles as modulatory gating mechanisms for information transfer between the cortex and subcortical structures such as the thalamus (Niedermeyer and da Silva, 1999), and may explain the dependence of imagery performance on these rhythms. Using transcranial magnetic stimulation (TMS), low amplitude oscillations were shown to be representative of an excitable state of the motor cortex (Sauseng et al., 2009). These findings indicate that the regulation of SMRs, specifically the mu rhythm, is likely to condition the cortex for forthcoming perception or action (Thut and Miniussi, 2009). A BCI that relies on changes in mu rhythm may be affected by pre-trial mu amplitude simply because high mu before the trial offers opportunity for larger decreases from baseline level. However, the ability to form motor imagery may also be modulated by oscillations representing motor network excitability.

Early work by Bishop demonstrated that the phase of ongoing cortical oscillations partially predicted the seemingly random variations in evoked potentials due to visual stimulation (Bishop, 1932). This demonstration led to the hypothesis that these oscillations represented cyclical cortical excitability. Later studies showed how these periods of excitability could be demonstrated by consistent changes in perceptual or motor thresholds. Phase and amplitude of the occipital alpha at the time of visual stimulus presentation has been shown to be predictive of stimulus detection (Niedermeyer and da Silva, 1999; Thut and Miniussi, 2009). Somatosensory cortical evoked responses to painful stimuli are also facilitated by the amplitude of 8–13 Hz mu motor rhythms (Ploner et al., 2006). Further evidence comes from the work of Kruglikov and Schiff (2003), who showed that auditory evoked potential morphology is a function of broadband EEG phase.

Visual evoked potentials (VEP)s are present in the scalp EEG as a result of the visual cue presented to the user in a synchronous BCI task. In motor-imagery tasks, this evoked response may be considered a source of artifact and removed or ignored in further processing. However, these evoked responses have been shown to be modulated by region of fixation (Hillyard and Anllo-Vento, 1998; Treder and Blankertz, 2010), and serve as a marker of visual attention. Visual attention is controlled by a distributed network of cortical and subcortical areas which act to provide “bias signals” that enhance or suppress the responses to visual stimuli (Hillyard and Anllo-Vento, 1998). The distinction between fixation and attention in the context of a BCI is an important one. In a cued BCI paradigm, attention is required for the subject to understand the cue and react to it, but fixation on that cue is not absolutely necessary (Treder and Blankertz, 2010). Of the components of the VEP, the N1, N2c, and P3b peaks have been shown to be influenced by visual attention (Niedermeyer and da Silva, 1999; Treder and Blankertz, 2010; Railo et al., 2011). Increases in amplitude of the first two components and a decrease in the latency of the P3b have been shown to be correlated with visual attention. Error-related potentials are also present in the EEG during BCI tasks. These evoked components of the EEG are generated after viewing feedback incongruent with the target response in both cursor movement and P300 tasks (Spüler et al., 2012; Iturrate et al., 2013). Although post-trial retraction based on error detection has been shown to improve BCI performance (Spüler et al., 2012), in this study, evoked potentials prior to the primary task were the focus.

The aim of this study was to identify intra-individual predictors of single trial BCI success. By finding brain signatures which correlate with improved performance, we can attempt to increase the accuracy and bit rate achieved with the interface. One possible way to do this is by gating trials with low predicted performance. By gating, we prevent classification from taking place if the signature of brain activity predicts poor future performance on the task. We evaluated the utility of three of these brain signatures, which we term “gating variables”: the amplitude and phase of ongoing motor rhythms, and the visually evoked potential produced in response to the cuing mechanism. We show that mu amplitude at the beginning of the trial, which is generally discarded during motor-imagery feature selection, is correlated with performance within a subset of subjects. Aside from one subject, the visual evoked potential did not associate with subsequent imagery performance, demonstrating that fixation to the cue does not modulate level of success on the task. Despite the potential for gating trials using mu amplitude, the utility of these features in an offline gating simulation was limited in its ability to produce an increase in device throughput.

## 2. Materials and methods

### 2.1. Experimental setup

A commercial EEG recording system (Guger technologies, www.gtec.at) was used to acquire data from subjects. Data were sampled at 256 Hz and band pass filtered at 0.1–30 Hz. This bandpass range was chosen to preserve VEP components and oscillations in the mu band. Data were recorded within the Simulink environment in MATLAB (version 2009b) and stored on a notebook computer (Dell Latitude E6400) running Windows XP. Subjects were seated in a chair facing an LCD monitor which displayed cuing and feedback information. The experimental protocol was approved by the Institutional Review Board of Penn State University.

### 2.2. BCI paradigm

Nine right-handed volunteers, all male with ages ranging from 18 to 37, participated in a cue-paced, one-dimensional center-out motor imagery task. Participants were recruited from the local area by means of a flier with no restrictions on gender. Channels FC3, FC4, C5, C3, C1, C2, C4, C6, CP3, CP4, P5, P3, P1, P2, P4, P6, PO3, and PO4 were recorded, in addition to three electrooculogram (EOG) electrodes placed on the lateral canthi as well as just above the nasion. All channels were referenced to linked earlobes, and ground was placed at Fpz. Each subject performed four sessions over a 2 week period. Each session lasted ~1.5 h.

During each session, the subject performed five runs of 60 trials each, divided equally between left, right, and no-target cues that were presented in a randomized sequence. Data from the first run of each session, the training run, was used to construct a classifier so the remaining four testing runs could be used to give feedback to the subject as they performed the task. After each feedback run, the classifier was updated with data corresponding the twenty additional trials of the left and right classes.

In each trial, the subject was cued by an arrow pointing in the left or right direction. Arrows were displayed symmetrically in the visual field to minimize asymmetry in the evoked potential due to cue type. This asymmetry, which can last up to 500 ms and is observable in C3 and C4 electrode locations over the motor cortex (Dias et al., 2010), can falsely contribute to BCI classification accuracy independent of user-driven modulation (Dias et al., 2009). Subjects were instructed to perform imagery of an object-oriented grasping action for the hand corresponding to the direction of the arrow being displayed. If no arrow appeared, the subjects were informed to relax and were given no visual feedback.

In each trial of the training run, a fixation cross would first appear at which time the subject would relax. If this were followed by an arrow cue 1 s later, the subjects were instructed to perform imagery while the arrow remained on screen for 2 s. Each trial was followed by a random inter-trial period of 1–2 s. During the testing runs, a target appeared on the far side of the screen in the direction of the arrow, and during the 2 s in which they performed imagery, a cursor moved on the screen to provide the subject with feedback.

The output of the classifier served as feedback which informed the subject how closely their EEG signals of intent matched templates for left and right cues developed in the training run. This was done by summing the squared distance *D* of the band power in trial *n* from the *m* ∈ (*left, right*) template band power over all time points *t* and features *f* (Equation 1). Each template μ consisted of mu and beta band powers from Laplacian-transformed channels C3, C4, P3, and P4 during the period of motor imagery, making up *f* = 8 total band power features. In the calculation of distance,

(1) $$\begin{array}{ll} D_{n,m}\left(t\right)=\overset{8}{\underset{f=1}{\sum }}{\left(x_{n,f}\left(t\right)-\mu_{m,f}\left(t\right)\right)}^{2}, & \; \end{array}$$

*x*_*n, f*_ represents a band power feature from a single trial. The feedback at each time point was calculated as the log ratio of the left distance over the right distance (Equation 2), as

(2) $$\begin{array}{ll} Feedback_{n}\left(t\right)=Feedback_{n}\left(t-1\right)+\text{log}\left(\frac{D_{n,left}\left(t\right)}{D_{n,right}\left(t\right)}\right). & \; \end{array}$$

This control algorithm drove the cursor to the left when this ratio was less than one, and to the right when the ratio was greater than one. Feedback was accumulated over the length of the trial and then reset to the center of the screen at the beginning of the next trial. *Z*_*n*_, the success of the subject at performing imagery during trial *n*, was defined according to Eqution (3),

(3) $$\begin{array}{l} Z_{n}=\{\begin{array}{ll} Feedback_{n}(T), & \text{if Cue}=right\\ -Feedback_{n}(T), & \text{if Cue}=left, \end{array} \end{array}$$

where the time index *T* marks the end of the trial. This Euclidean distance-based classifier, unconventional in the LDA- and SVM- dominated field of BCI, was used due to a previous study which compared the performance with conventional classifiers (Geronimo et al., 2011). Over the four sessions, each subject completed 16 test runs consisting of 960 trials, with 320 trials each for left, right, and no-target cues. The data belonging to the roughly 320 trials of left and right cues were analyzed offline using the methods described below.

### 2.3. Artifact correction

The first step in offline data preprocessing was removal of eye-related artifacts that resulted from blinking or eye movement. This was a two-step process including artifact reduction and trial rejection. Artifact reduction was accomplished by linear regression (Schlögl et al., 2007). This least-squares method assumes the linear superposition of neural and artifact sources to produce the measured signal. Assuming the independence of the artifact sources and the neural sources, data can be used to find a weight matrix, which represents the projection of noise sources onto neural sources. We used the 18 EEG channels as our recorded signal **Y**, and the three EOG channels as the noise sources **U**, to find the weight matrix **W**, and solve for decontaminated neural sources **S** (Equation 4).

(4) $$\begin{array}{l} {\text{Y}}_{18\times T}={\text{S}}_{18\times T}+{\text{W}}_{18\times 3}\cdot {\text{U}}_{3\times T}. \end{array}$$

Here, *T* is the length of the data segment from which the weight matrix is calculated. In practice, this method may be suboptimal if there is significant leakage of the task-relevant EEG data into the designated noise channels, rendering the assumption of independence void. To minimize this effect, we solved for **W** using data which was sampled from when the EOG channel exceeded 75 μV, as in the case during a blinking event.

Following artifact reduction, trials were rejected if channel FC3 displayed absolute amplitude of greater than 50 μV. To control for artifact in unintentional movements, bipolar electrodes were placed on the forearms of a subset of the subjects to record electromyographic (EMG) muscle activity during the recording session. EOG and EMG were both analyzed offline to rule out possible contamination from overt eye and arm movements.

### 2.4. Extraction of gating variables

#### 2.4.1. Amplitude and phase of peri-stimulus mu

Data in channels C3 and C4 were filtered with a Laplacian spatial filter to localize the mu rhythms specific to the motor cortex (McFarland et al., 1997). Peak mu frequency in these channels was found for each subject using the multitaper spectral analysis method (Mitra and Bokil, 2008). The EEG data in these channels were filtered using a zero-delay filter in a 2 Hz range around the peak mu frequency. A Hilbert transform was applied to this band-limited signal to generate the analytic signal *S*_*a*_(*t*), comprising a real part *S*(*t*) made up of the original data, and an imaginary part *H*(*t*), its Hilbert transform (Equation 5),

(5) $$\begin{array}{l} S_{a}\left(t\right)=S\left(t\right)+iH\left(t\right). \end{array}$$

The amplitude of the signal *A*(*t*) was calculated as the Euclidean norm of the real and imaginary parts of the analytic signal (Equation 6).

(6) $$\begin{array}{l} A\left(t\right)=\sqrt{S^{2}\left(t\right)+H^{2}\left(t\right)}. \end{array}$$

The instantaneous phase ϕ(*t*) was calculated as the four quadrant inverse tangent of the ratio of the imaginary part of the analytic signal to the real part (Equation 7). Phase ranged from −π to π.

(7) $$\begin{array}{l} \phi \left(t\right)=\arctan\frac{H\left(t\right)}{S\left(t\right)}. \end{array}$$

**Figure 1:**
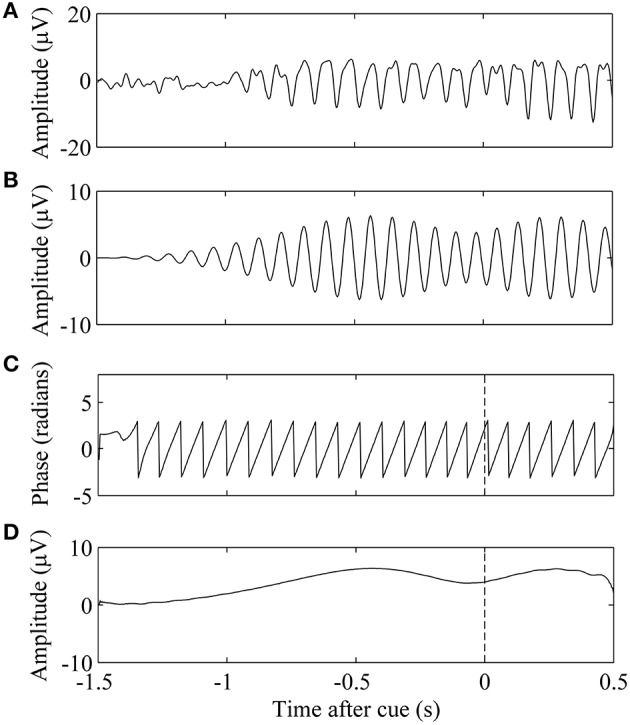
**Phase and amplitude extraction of a single EEG trial**. **(A)** Trial *n* of EEG data. **(B)** Signal was filtered in the subject-specific mu band. **(C)** The phase of the band-limited signal at τ_*n*_, the time of cue presentation, is marked by the vertical dashed line. **(D)** Amplitude of the band-limited signal.

For each trial *n*, the amplitude and phase of mu in C3 and C4 at the time of cue presentation were extracted (Figure 1). Mu amplitude was the first gating variable. Linear regression between trial success and amplitude was used to determine association. We assumed that the success at the end of the trial was a linear function of the natural-log-transformed peri-stimulus mu amplitude (Equation 8). Log transformation was performed on the spectral features to enforce the normality of the data as well as reduce outlier effects (Gasser et al., 1982).

(8) $$\begin{array}{l} Z_{n}=\alpha \times \text{ln}\left(A\left(\tau_{n}\right)\right)+\beta . \end{array}$$

In this equation, α and β are the parameters of the regression, and *A*(τ_*n*_) is the magnitude of the mu rhythm at the time of the cue of trial *n*. We used the slope of the regression α, as the measure of correlation between mu amplitude and trial success.

Mu phase was the second gating variable. We assumed trial success was a cosine function of the phase of the mu rhythm.

(9) $$\begin{array}{l} Z_{n}=\gamma \times \cos\left(\phi \left(\tau_{n}\right)-\delta \right)+\epsilon . \end{array}$$

Here, γ, δ, and ϵ are regression parameters specifying the amplitude, phase, and offset of the fitted cosine, and ϕ(τ_*n*_) represents the phase of the mu rhythm at the time of the cue of trial *n*. We specified the frequency of the cosine to be one cycle per 2π of phase. To find the parameters of this cosine model, we performed a non-linear regression using the MATLAB function *nlinfit.m*, with initial parameter estimates of γ, δ, and ϵ as 1, π, and 0. The correlation measure between mu phase and trial success was the amplitude of this fitted curve, γ.

#### 2.4.2. Match to VEP template

The final gating variable was related to the quality of the VEP. In order to define whether the subject produced an evoked response during a single trial that represented good fixation, we needed to define a template that represented a typical VEP waveform for that subject.

**Figure 2:**
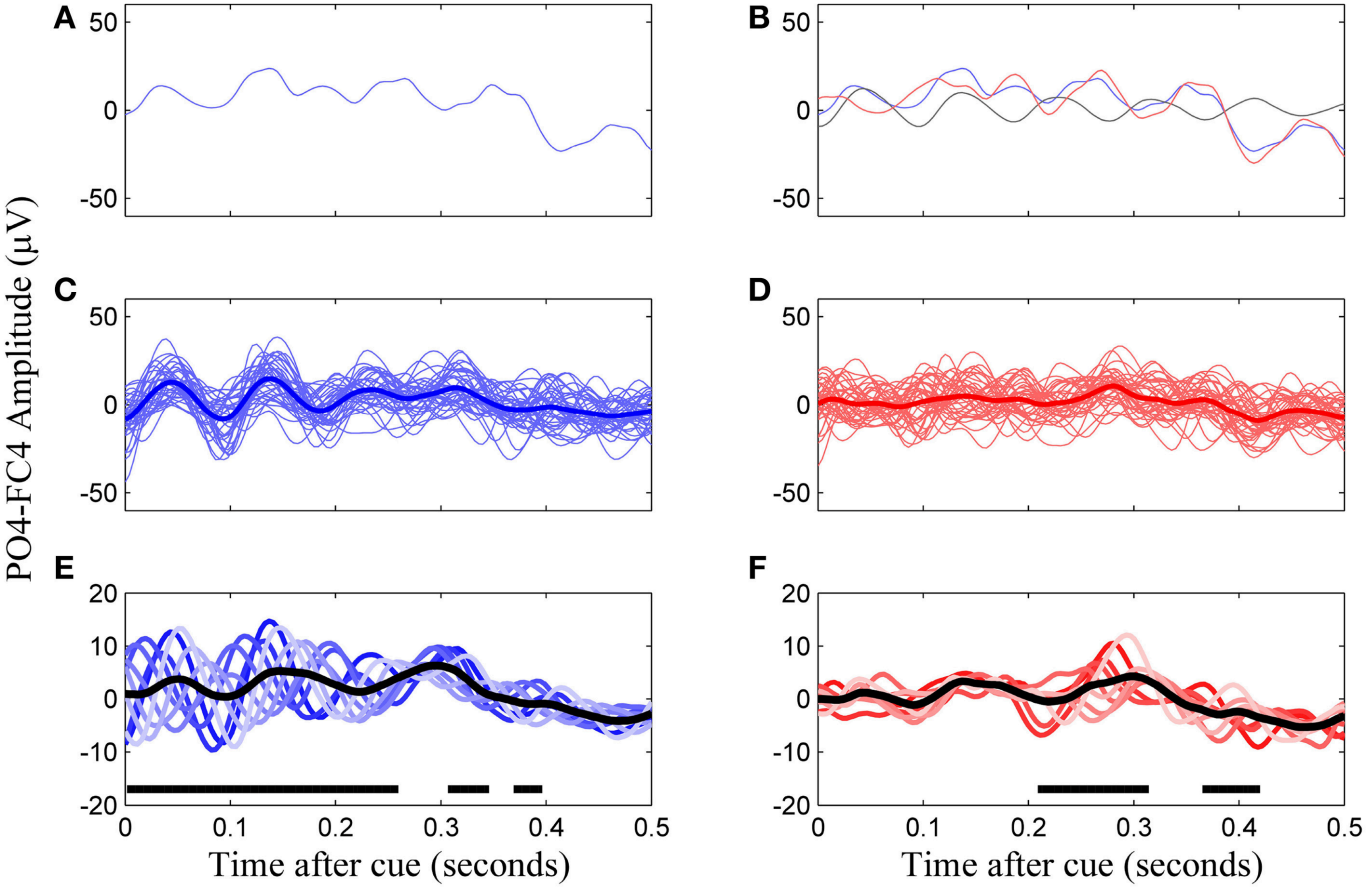
**Construction of the VEP template from phase-corrected left cue trials, channel PO4-F4, subject 8**. **(A)** A single trial of VEP data. **(B)** Phase-matched correction by subtraction of a non-cued trial in the same phase group (falling within a same 45°window, black line) results in the phase-corrected trial (red line). **(C)** Uncorrected trials of the same phase group and their average. **(D)** Corrected trials of the same phase group and their average. **(E)** All eight phase groups and the average resulting VEP (black trace). Regions with thick horizontal lines indicate times during which the mean VEPs between the eight phase groups were significantly different as determined by ANOVA (*p* < 0.05, Bonferroni corrected). **(F)** The final template for this subject is the average of the phase corrected trial groups (black line).

**Figure 3:**
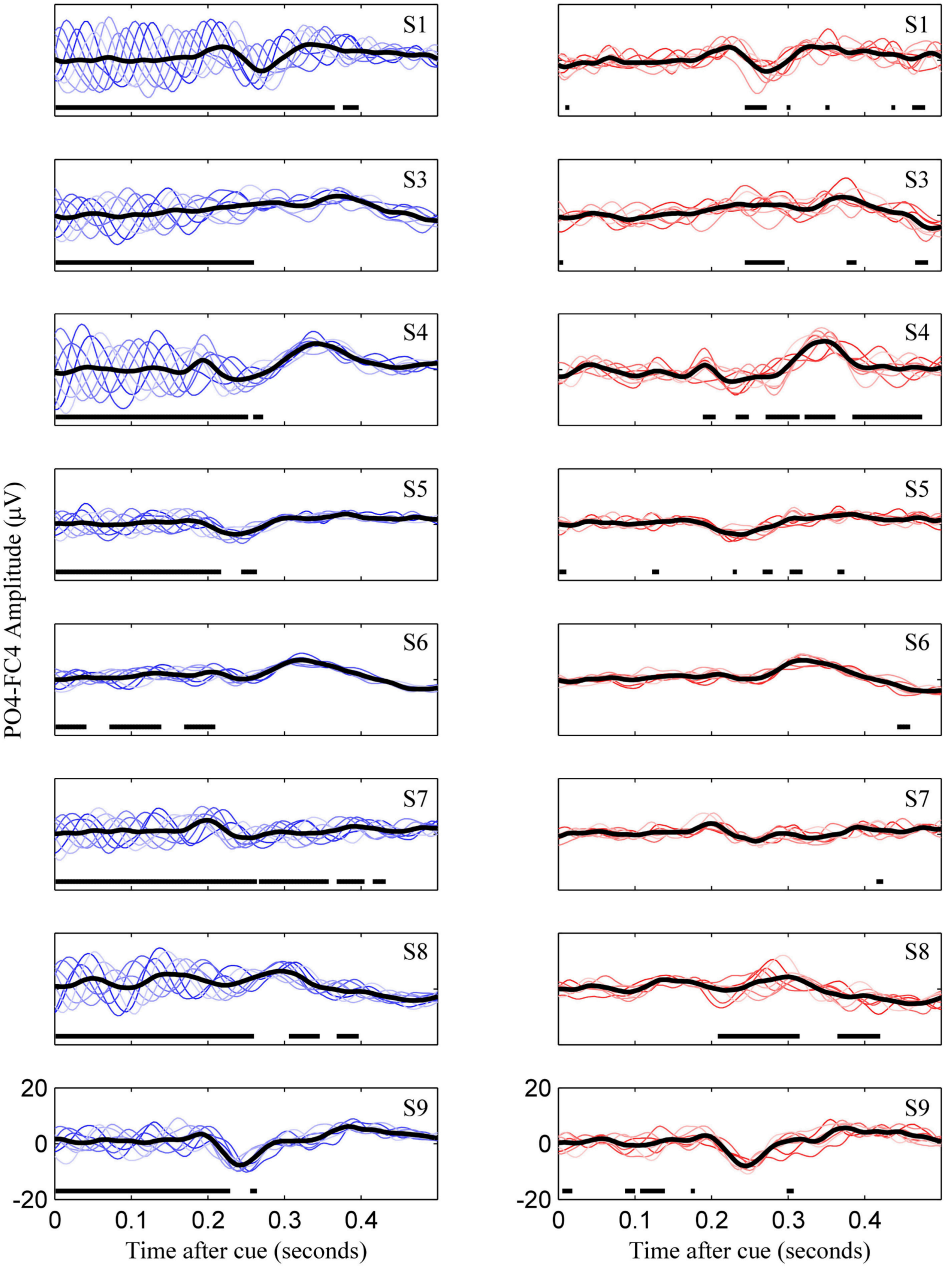
**Uncorrected (left) and phase match corrected (right) VEPs for all subjects**. This represents for all subjects what is Figures 2E,F for subject 8. Colored lines are the eight phase groups, and the black trace is the resulting average VEP. Regions with thick horizontal lines indicate times during which mean VEPs between the eight phase groups were significantly different as determined by ANOVA (*p* < 0.05, Bonferroni corrected).

Following 0.1–30 Hz bandpass filtering, we first performed phase-matched control trial correction, following the methodology defined by Kruglikov and Schiff (2003). This was done because the phase of EEG at the time of the cue presentation introduces an averaging bias. Individual trials were corrected using phase-matched control trials, or trials in which a similar alpha phase was evident but in which there was no stimulus presented. By subtracting out the average of control trials of the same phase group, defined in 45°phase bins, we remove the predominant alpha rhythm and are left with a trace that better reflects the underlying neural correlate of the VEP (Figure 2). Such a procedure removes peri-stimulus bias by phase of the evoked potential in all subjects (Figure 3).

Once corrected for phase, templates for left and right cues were defined by averaging EEG data over trials corresponding to each cue type for bipolar channels PO3-FC3 and PO4-FC4. This referencing scheme was the closest approximation to the standard Oz-Fz (Odom et al., 2010) we could accomplish with our electrode montage. The timing of the template ranged from 100 to 400 ms after cue presentation.

A metric describing the match of individual trials to the template was created using a matched filter approach. This technique comes from radar communication as a method for maximizing the probability of detecting a target waveform in the presence of Gaussian noise (McClellan and Purdy, 1978). Although a derivation can be found elsewhere (Levanon and Mozeson, 2004), the optimal filter for maximizing the signal to noise ratio of a signal generated by a linear, time invariant system with added Gaussian noise is the time-reversed version of the transmitted signal. This time-reversed template is called the matched filter. We applied the template as the transmitted signal *s*(*t*), and the phase-corrected single trial data as *r*_*n*_(*t*). The output of the matched filter *y*_*n*_(*t*) was the convolution of the trial data with the time-reversed template (Equation 10). The output of the matched filter that we used was the central value of this convolution, normalized to the magnitude of the template (Equation 11). This value, which we called the matched filter value (MFV), was related to the signal to noise ratio of the VEP, and served as the third gating variable. Trials with large MFV were interpreted as being trials which the subject produced a robust VEP.

(10) $$\begin{array}{r} y_{n}\left(t\right)=r_{n}\;\left(t\right)*s\left(-t\right). \end{array}$$

(11) $$\begin{array}{r} MFV_{n}=\frac{y_{n}\left(0\right)}{\left||s|\right|}. \end{array}$$

Here, ^*^ represents the convolution function, and *y*_*n*_(0) is the central value of the output of the convolution. The magnitude of the template *s*, in this case the Euclidean norm, is denoted by vertical brackets.

Similarly to the previous gating variables, we determined the relationship between the MFV and success on the BCI task. Again, we assumed that the trial success was a linear function of the MFV (Equation 12).

(12) $$\begin{array}{l} Z_{n}=\zeta \left(MFV_{n}\right)+\eta . \end{array}$$

The slope of this regression, ζ, reflects the correlation between of the outcome of the trial and the match to a VEP template of good fixation.

### 2.5. Permutation testing for significance

The three gating variables were tested for significant correlation with trial success in each subject. Because the dependent variable, the trial success, was not normally distributed, we chose to perform a non-parametric permutation test to determine the statistical significance of the regression parameters. We first computed the test statistic *Q*_*obs*_, the slope of the linear regression (α, ζ) or the amplitude of the fitted cosine (γ). Then we shuffled the *Z* success outcomes between the trials and used the same fitting procedures to calculate the permuted statistics, *Q*(*k*) for each permutation *k* = 1…*K*, where *K* = 1000.

This permutation was performed with each gating variable from EEG features in two channels. Correction for multiple comparisons was done for each hypothesis test in the following manner, as described in more detail in Lage-Castellanos et al. (2010). For each permutation *k*, both the maximum *Q*_*max*_(*k*) and minimum *Q*_*min*_(*k*) values were chosen from the two *Q*(*k*) statistics evaluated for both channels. This resulted in *K*-values making up each of the maximum and minimum empirical distributions of the randomized data set. The calculation of p-statistics, the probability of *Q*_*obs*_ belonging to the null distribution of the permuted set, is given in Equation (13).

(13) $$\begin{array}{ll} p_{high}=\frac{\sum_{k=1}^{1000}\left(H\left(Q_{max}\left(k\right)-Q_{obs}\right)\right)+1}{1000+1}. & \; \end{array}$$

**Figure 4:**
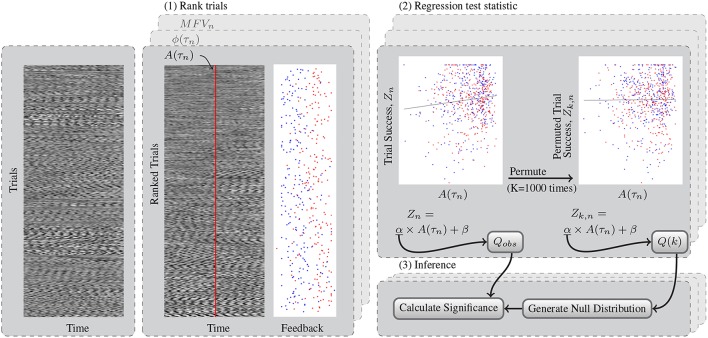
**This flowchart shows a generic example of the permutation test for determining significant relationships between gating variables and trial success**. (1) Test data from left and right trials are sorted using the rankings determined by each gating method. Shown here, the trials are ranked according to mu amplitude at the time of the cue, *A*(τ_*n*_). Trials with high *A*(τ_*n*_) are shown on the bottom of the block. The feedback at the end of these trials is also shown, with left trials in blue and right trials in red. Feedback was subsequently simplified to trial success, *Z*. (2) The observed test statistic, *Q*_*obs*_ is the slope of the regression that relates mu amplitude to trial success. The permuted test statistic *Q*(*k*) was computed *K* = 1000 times after permuting the success scores of the trials to *Z*_*k, n*_. (3) The *k*^*th*^ value for the maximum and minimum null distributions *Q*_*max*_ and *Q*_*min*_ were found from *Q*(*k*) across both channels tested. Finally the *p*-value of the test statistic distribution is computed as the percentage of values in this null distribution exceeding that of the observed test statistic.

Here, H is the Heaviside function. Ones are added to the numerator and denominator to include the *Q*_*obs*_ in our null distribution. *p*_*low*_ was also calculated similarly using *Q*_*min*_ to test for significance from both tails of the null distribution. Findings of either *p*_*high*_ or *p*_*low*_ < 0.025 are significant deviations from chance. The generic process of trial ranking and permutation testing for significance is shown Figure 4.

### 2.6. Trial gating simulation

To simulate the effect of utilizing the potential gating variables online, we computed how the accuracy and bit rates would change for each subject if a portion of the trials having the least predictive value for task completion were removed. This predictive value was determined from the output of a linear discriminant analysis (LDA) classifier, which had been trained with the gating variables derived from channels in the right and left hemispheres as the input and trial success as the outcome variable. This simulation was run using individual gating variables determined to have significant predictive value by the permutation test, as well as with a combination of these gating variables. Trials in the test set that were classified as having the lowest predictive value were gated, meaning they were stopped before the imagery period began. The simulation was repeated at different thresholds, from gating no trials up to gating 70% of the total trials. Gated trials were skipped 500 ms after stimulus presentation and were interpreted as no-decision while taking 1.5 s (1 s before cue and 0.5 s after) to complete. Allowed trials resulted in a decision while taking 4 s to complete. Accuracy was defined as the number of allowed trials performed correctly over the total number of allowed trials.

Four-fold cross validation was used to find an average of the accuracy of the gating procedure at each threshold. In each fold of the classification scheme, a single session was used as the test set, while trials from the remaining sessions were used to train the classifier. The accuracy was calculated as the number of trials completed correctly out of the total number of trials attempted after gating. The bits per trial, *B*, was calculated for an *N* = 2 choice task having accuracy *P* following the form of Equation (14) from Wolpaw et al. (2002).

(14) $$\begin{array}{r} B={log}_{2}N+P{log}_{2}\;P+\left(1-P\right){log}_{2}\frac{\left(1-P\right)}{\left(N-1\right)}. \end{array}$$

The bit rate *BR* in bits/min was calculated using Equation (15).

(15) $$\begin{array}{r} BR=B∕T_{\mu }, \end{array}$$

where the average trial time in minutes, *T*_μ_, was increased to represent a penalty for disposing trials. *T*_μ_ was calculated from the number of allowed trials, *N*_*a*_, and the gated trials, *N*_*g*_, using Equation (16).

(16) $$\begin{array}{r} T_{\mu }=\left(4N_{a}+1.5N_{g}\right)∕60N_{a}. \end{array}$$

Significant differences in the baseline vs. gated accuracies and bit rates were calculated by a non-parametric Wilcoxon rank sum comparison of means over the four cross-validation folds.

## 3. Results

### 3.1. BCI performance

The operation of the BCI was successful for eight of the nine subjects, whose online accuracies for each session are given in Table 1. Subject 2, with an average accuracy of 50.4%, indicative of random BCI control using this method, was not considered successful at operating the BCI system. Because we cannot be sure there are any signals relevant to motor imagery produced by this individual, they were omitted from further analysis. Subject 3 showed a significant learning effect, with others displaying a smaller or non-existent effect. LDA with 10-fold cross validation was performed for features derived from EOG and EMG channels. Mean classification accuracy for all eight subjects using EOG features was 51.7 ± 2.9% and mean classification accuracy for the four out of eight subjects with recorded EMG features was 49.2 ± 0.8%. Bounds are standard deviations.

**Table 1:** **Online performance**.

	**S1**	**S2**	S3^*^	**S4**	S5^*^	S6^*^	S7^*^	**S8**	**S9**	**Grand Avg**.
Session 1	79.4	53.6	63.2	83.7	81.5	51.3	77.5	73.1	95.6	73.2
Session 2	74.1	44.6	72.1	85.3	66.0	69.6	60.9	74.8	98.1	71.7
Session 3	86.5	55.2	81.5	84.6	65.4	72.3	56.7	85.9	98.1	76.3
Session 4	83.8	48.4	87.4	93.0	65.2	63.1	74.8	74.4	99.4	76.6
Total	80.9	50.4	76.1	86.7	69.4	64.1	67.5	76.9	97.8	74.4

Mean accuracies (% trials correct) of online classification over the four runs of each session for each subject. Four subjects marked by asterisks had performances which significantly differed by session, as determined with a chi-squared test for independence.

### 3.2. Mu amplitude and VEP correlation associate with trial success

**Figure 5:**
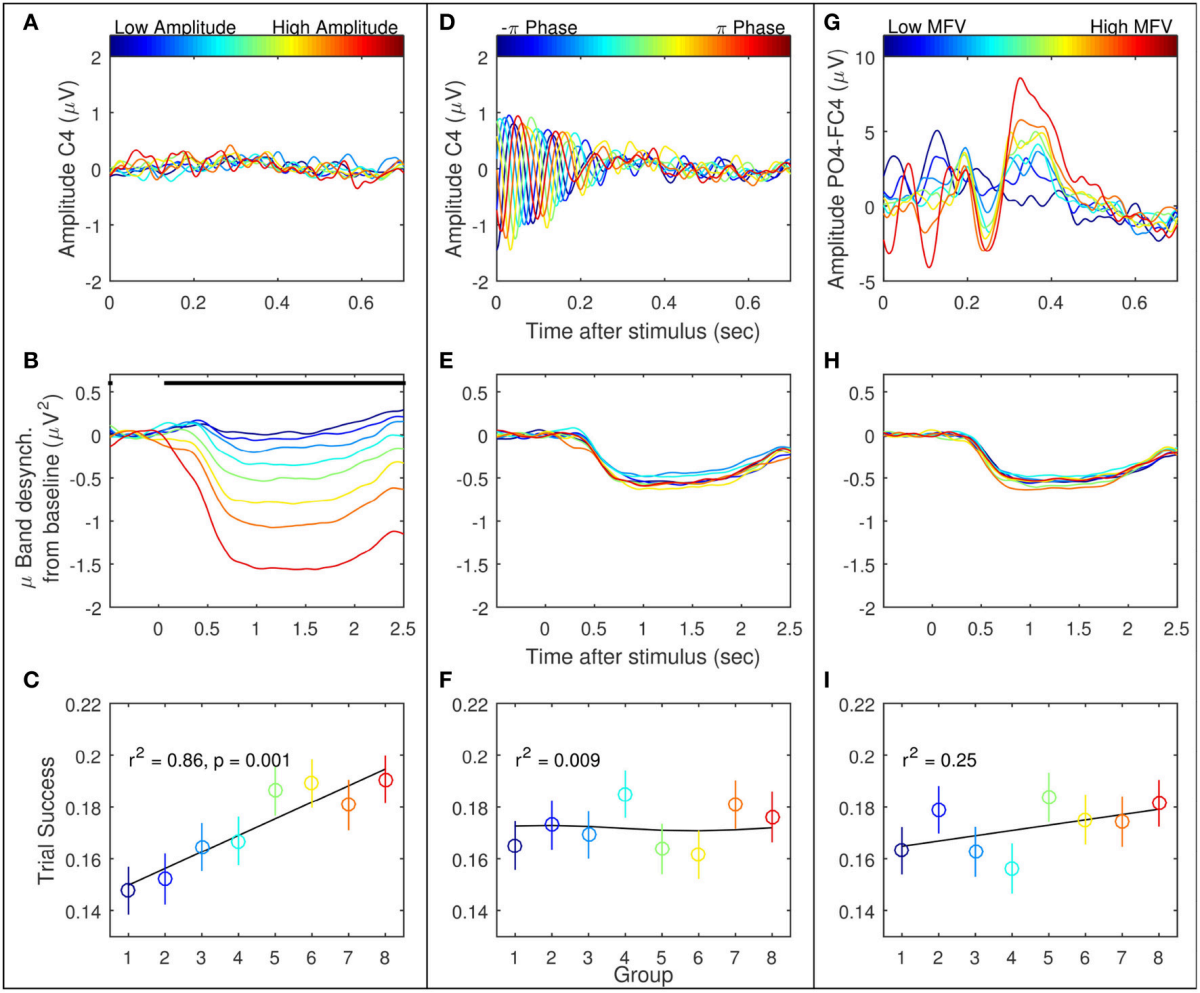
**Three EEG features were considered as potential gating variables (shown here are the right hemisphere features): mu amplitude in C4 at stimulus presentation (left column, A–C), mu phase in C4 at stimulus presentation (middle column, D–F), and match to PO4-FC4 VEP template (right column, G–I)**. For each feature, all recorded trials were divided into eight groups, from low feature value to high, represented by the eight colored lines/points. The first row is the average of the EEG in these groups. The second row is the average mu suppression from baseline in C4 in these groups. The last row is the mean and standard error of the success across all trials by group.

**Figure 6:**
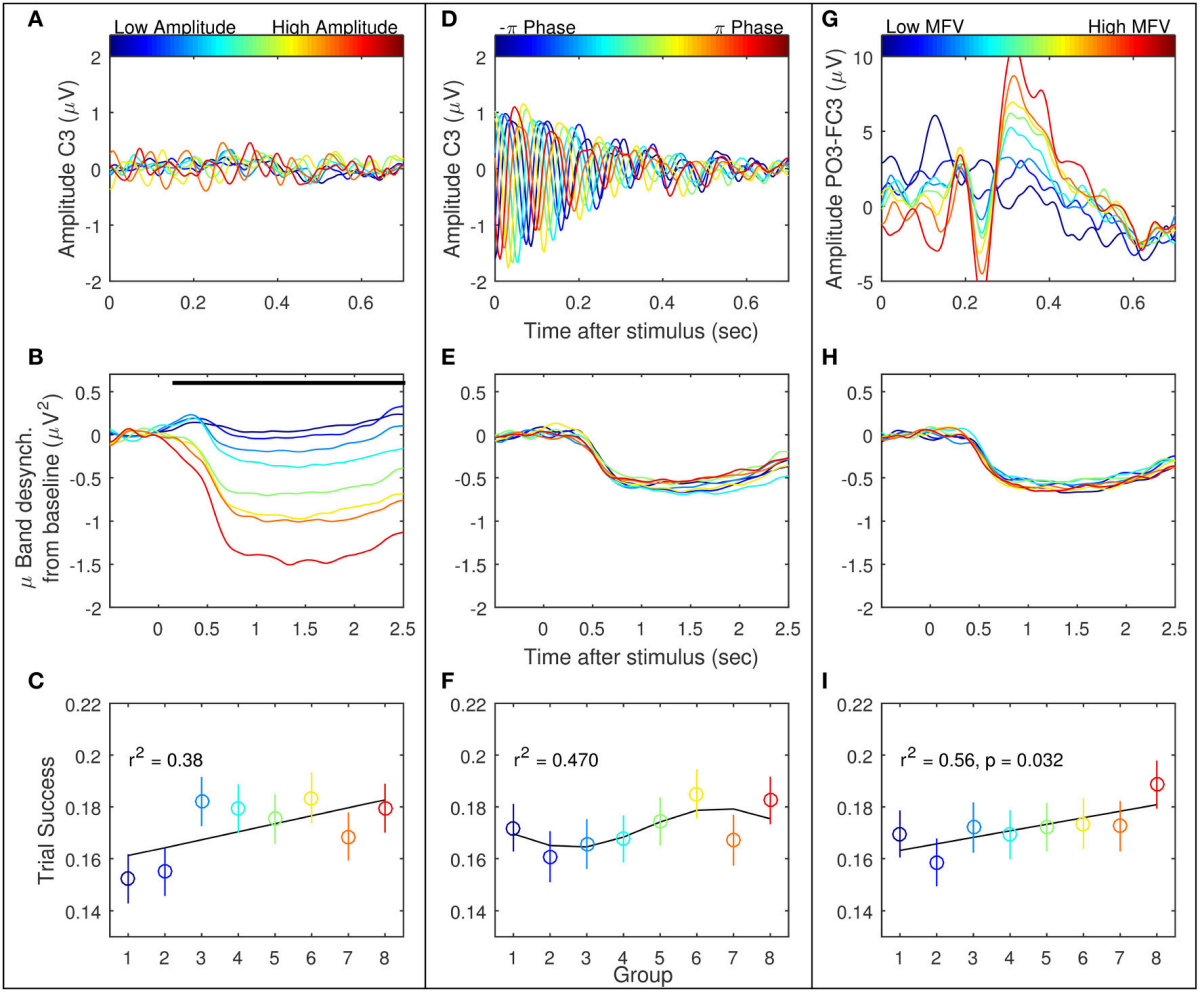
**Three EEG features were considered as potential gating variables (shown here are the left hemisphere features): mu amplitude in C3 at stimulus presentation (left column, A–C), mu phase in C3 at stimulus presentation (middle column, D–F), and match to PO3-FC3 VEP template (right column, G–I)**. Refer to Figure 5 for further description.

Trials were ranked according to the three gating variables of mu amplitude, mu phase, and MFV. Subject-specific mu frequencies for Laplacian-derived channels C3 and C4 are given in Table 2. Figure 5 shows the grand average results for the study in right hemisphere channels, in which trials are subdivided into eight groups based on ranking by each gating variable. Average EEG traces for each group are shown in subplots (a), (d), and (g), while mu suppression from baseline is given in the second row in subplots (b), (e), and (h). For groups ranked by baseline mu amplitude, there is a significant difference in the level of suppression during imagery (Figure 5B, black bar indicates ANOVA with *p* < 0.05, Bonferroni corrected for 768 time points). MFV shows a positive association between gating variable group and trial success (Figure 5I, *r*^2^ = 0.25, not significant), mu phase shows no association, and the positive correlation between trial success and mu amplitude is significant (Figure 5C, *r*^2^ = 0.86, *p* = 0.001). Positive correlations between trial success and both mu amplitude and MFV are also evident in the left hemisphere channels (Figure 6), of which the correlation between MFV and trial success is significant (Figure 6I, *r*^2^ = 0.56, *p* = 0.032).

**Table 2:** **Subject specific mu frequencies**.

**Channel**		**C3**	**C4**
Subject	1	11.75	11.75
	3	10.75	10.50
	4	10.25	10.00
	5	11.00	10.75
	6	13.00	13.00
	7	13.00	13.00
	8	10.50	10.50
	9	12.25	12.00

Subject-specific center frequencies (in Hz) of the mu bin in channels C3 and C4.

On an individual basis, the permutation test procedure determined that subjects 4, 6, 8, and 9 produced in at least one channel a slope of regression, α, which was significantly greater than that produced by the random null distribution, indicating a significant positive correlation between mu amplitude and motor imagery task success (Table 3). Non-linear regression produced no significant cosine fits between mu phase and trial performance. Finally, only subject 9 displayed a significant correlation between the MFV gating variable, with high values of the gating variable in both channels PO3-FC3 and PO4-FC4 corresponding to greater trial success.

**Table 3:** **Results of the permutation test with the three gating variables**.

**Feature**		**Amplitude (α)**		**Phase (γ)**		**MFV (ζ)**	
**Channel**		**C3**	**C4**	**C3**	**C4**	**PO3-FC3**	**PO4-FC4**
Subject	1	0.012	0.013	0.010	0.011	−0.0001	0.0000
	3	0.003	0.008	−0.016	−0.011	0.0004	0.0005
	4	0.016	**0.044**	0.006	−0.009	−0.0002	−0.0003
	5	−0.017	0.003	0.013	−0.006	0.0000	0.0002
	6	**0.029**	**0.055**	−0.012	0.019	0.0001	−0.0003
	7	−0.001	0.011	−0.007	−0.011	0.0003	0.0002
	8	0.013	**0.027**	0.021	0.013	0.0003	0.0004
	9	**0.018**	0.012	0.015	−0.006	**0.0011**	**0.0007**

Values in the six columns represent value of Q_obs_, or the slope/amplitude parameters of the three regressions in two channels. Bolded values indicate a significant Q_obs_ at the 0.025 level.

### 3.3. Trial gating simulation

**Figure 7:**
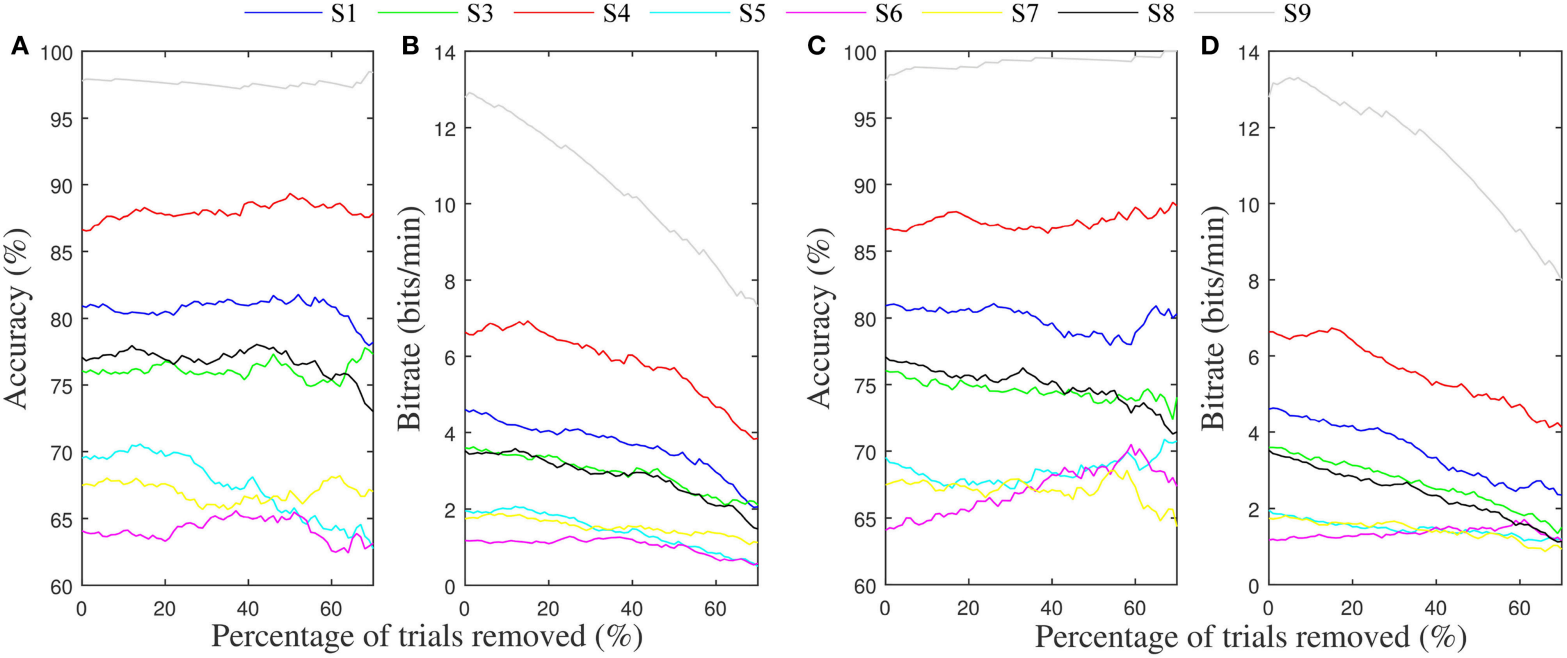
**Average accuracy and bit rates from the four-folds of the gating simulation**. **(A)** Average accuracy achieved for each subject after gating up to 70% of trials randomly. **(B)** Resulting bit rate. **(C)** Average accuracy achieved for each subject after gating of trials up to 70% based on a linear combination of mu rhythm amplitude and MFV features. **(D)** Resulting bit rate.

Table 4 gives the average of the maximum accuracies and bit rates achieved by each subject in the four-fold simulation procedure of gating with the potential gating features. In most instances where the permutation test indicated a subject would benefit from gating trials of low value, the simulation of offline gating yielded an increased accuracy. Bolded values show where the gated accuracy or bit rate of the four-folds of the simulation procedure were significantly larger than the non-gated values (Wilcoxon rank sum test, *p* < 0.05). The only significant increase in accuracy was seen for subject 9, where gating ~60% of trials based on the match to VEP resulted in improvement to 100% accuracy. Figures 7C,D show how the accuracy and bit rate change for each subject as a function of the number of trials that are gated based on a combination mu amplitude and MFV. In comparison, Figures 7A,B show how the accuracy and bit rate are affected by gating of random trials.

**Table 4:** **Simulated gating results**.

**Subject**	**Acc_o_ (%)**	**BR_o_ (bits/min)**	**SMR amplitude**		**MFV**		**SMR Amp + MFV**	
			**Acc_max_ (%)**	**BR_max_ (bits/min)**	**Acc_max_ (%)**	**BR_max_ (bits/min)**	**Acc_max_ (%)**	**BR_max_ (bits/min)**
1	80.9	4.61	82.0	4.65	81.2	4.51	81.1	4.64
3	76.1	3.61	77.8	3.63	76.6	3.64	76.0	3.60
4	86.7	6.66	88.6	6.71	88.1	6.63	88.6	6.73
5	69.4	1.92	70.7	1.95	72.9	2.19	70.9	1.87
6	64.1	1.18	70.9	1.81	64.8	1.13	70.5	1.71
7	67.5	1.75	68.5	1.78	67.9	1.73	68.6	1.81
8	76.9	3.47	77.6	3.44	76.7	3.40	76.8	3.44
9	97.8	12.79	98.7	12.71	**100.0**	13.41	**100.0**	13.31

Simulated gating using two gating variables and their combination. Columns Acc_o_ and BR_o_ give the accuracy and bit rate for the online operation of the device. Acc_max_ is the maximum average accuracy over the four-folds of the simulation procedure after a percentage of trials up to 70% were gated 500 ms after cue presentation. BR_max_ gives the maximum average bit rate due to the same gating. Bolded values mark where a significant improvement in accuracy or bit rate was achieved by the simulation.

## 4. Discussion

### 4.1. Prospects for gating features

In BCI, single trial identification of user intent is a difficult task; nevertheless, we have shown that ongoing oscillatory activity of the motor cortex contains additional information that co-varies with motor-imagery task performance. Of the three features explored, the technique of selecting for trials with high mu amplitude was the most consistently correlated with trials in which users are most likely to achieve higher performance.

The topic of oscillatory electrical brain phenomena influencing motor excitability is controversial (Mäki and Ilmoniemi, 2010), but the evidence is stronger for cortical excitability changes to be correlated with SMR amplitude than with phase. The lack of correlation between the phase of the mu rhythm and subsequent suppression efficacy is in agreement with other studies which found that phase had little effect on mu suppression (Mäki and Ilmoniemi, 2010). Whereas, the phase at the time of cue presentation did have an effect on early evoked potential morphology (Figure 2E), it is not surprising that the effects generally do not propagate 1–3 s after the cue, during which mu suppression is evident. On the other hand, all subjects displaying significant correlation between mu amplitude and BCI task success performed better on individual trials when the amplitude of mu was high over channels C3 and C4 at the onset. These results build upon the those of Blankertz et al. (2010), who demonstrated that baseline SMR amplitude distinguished good and poor performers, and Maeder et al. (2012), who showed the same effect of SMR amplitude on BCI performance *within* subjects.

The relationship between pre-stimulus SMR amplitude and performance could be due to two reasons. Our classifier was based on reduction of mu from a baseline level to an attenuated level, and as a result, trials with larger baseline mu amplitude have a greater potential for mu power reduction. In addition, the amplitude of the mu rhythm reflects the state of excitability of its generating network (Mäki and Ilmoniemi, 2010). While our finding is in disagreement with studies that point to a desynchronized cortex as one primed for motor output (Sauseng et al., 2009; Mäki and Ilmoniemi, 2010), Blankertz et al. (2010) showed that a successful predictor of imagery performance across subjects was an increased resting state mu rhythm. In a follow up study, they showed that this performance increase was associated with larger recruitment of motor and premotor regions. They concluded that recruiting more synchronized neurons for motor imagery led to higher resting mu amplitude as well as higher performance (Halder et al., 2011). In our study we show that not only is this relationship between resting mu amplitude and imagery performance valid between subjects, but also on a single-trial basis within subjects.

The third tested gating variable did not produce consistent evidence to warrant widespread use as a metric for predicting motor-imagery success, although the potential for this was evident in one user. Risner et al. showed that, following phase-matched correction of VEP data using unstimulated controls, average VEPs in different phase groups display the same VEP morphology (Risner et al., 2009). We also found this to be the case for early evoked potentials, although after phase-matched control trial correction, there was some phase dependence in later evoked potentials in certain individuals (Figure 3, subjects 4 and 8). Because the MFV was calculated from phase-corrected VEP data, the value of this metric can be interpreted to be free of oscillatory alpha bias and instead can be associated with attention to the visual task. However, using a subject-specific VEP template to define the MFV, we were unable to find a consistent correlation between this marker of attention and the subsequent modulation of motor rhythms. Although subject 9 did display a relationship between high MFV and good performance, overall there was no group-wide trend. This finding has important implications for BCI research. It demonstrates that fixation to the visual cue is not critical for task success in most users. For these users, control over the BCI communication device should not be limited by their ability to directly fixate with cues on the screen, a critical allowance in the case of severe oculomotor impairment that could occur in stroke or late stage neurodegenerative disease.

### 4.2. Trial gating simulation

The goal of this study was to identify certain brain signals present during a motor imagery BCI experiment that could be used as a supplementary control mechanism to improve accuracy, speed, and even allow for asynchronous timing.

To show this might be possible, we attempted to use these signals to predict trials with low probability of successful completion, and gate them so that they aborted before finishing. In the majority of cases where a correlation between the gating variables and trial success was present, offline simulation of the gating procedure failed to produce increases in accuracy or bit rate for the motor imagery BCI system. The notable exception was seen in subject 9, where significant increases in accuracy were accomplished by discarding trials with low MFV. Because this user was already quite high performing, throwing away 60% of the trials to increase accuracy from 97 to 100 percent was detrimental overall to the information transfer rate of the BCI (Figures 7C,D).

In all other cases, the gating procedure did yield improvements to system accuracy, but these increases were not significantly above the baseline scenarios in the cross-validation procedure. Furthermore, the gains in accuracy were not great enough to overcome the extra time it takes to gate a trial, resulting in slower system speed. Utilizing a combination of gating features as part of the gating system also resulted in improved performance for subject 9, although the level of improvement was less than that resulting from gating by MFV alone. In fact, for most subjects, gating with a combination of multiple features was less effective than use of individual variables that correlated with BCI performance. This demonstrates that for a gating system such as this to be successful, the mechanism of gating must be personalized to the user based on brain signatures that correlate with performance.

The discrepancy in results between the permutation procedure and the gating simulation highlights the distinction between what we are looking for in our study, specifically, a correlation between pre-task EEG features and task performance, and the use of this knowledge in an online BCI. We show that this type of gating is successful for a number of subjects to improve accuracy on the motor imagery task. However, the increase in accuracy needs to be substantial for this method to be useful.

### 4.3. Study limitations

Although the oscillation of cortical rhythms is associated with the depolarization and hyperpolarization of large groups of neurons, at the level of EEG the linkage with motor cortical excitability may be tenuous; at this level of measurement from scalp, both excitatory and inhibitory neurons contribute to oscillatory behavior (MacKay, 1997). Consequently, broadly recorded signatures of rhythmic activity may be unable to describe excitability in a local group of motor neurons responsible for hand imagery. This is especially true when we attempt to use the excitability of the motor region as a predictor of SMR modulation that occurs on the order of 500–1000 ms later.

The lack of findings for the MFV gating variable could be due to poor characterization of individuals' VEPs. The recording parameters included a 0.1–30 Hz bandpass filter, which is narrower than the clinical recommendation of 1–100 Hz for identifying individual VEP peaks (Odom et al., 2010). As a result, these peaks may have undergone some attenuation and blurring, and may not have been the ideal template for assessing attention with small variations in peak amplitude.

A substantial limitation of our study is the offline analysis of BCI data. Feedback is an integral part of the BCI system, and we cannot assume that the user will respond similarly when faced with a scenario where trials are gated on a regular basis. Just as poor feedback on a trial may have led to subsequent good performance on the following trial, we might expect a gated trial to produce increased vigilance or promote focus for the upcoming trial. This is a limitation that cannot be reconciled by offline analysis; these effects need to be quantified in a real-time adaptive scenario in a future study. What remains to be seen is how the feedback of the gating procedure affects the stationarity of these signals, and whether trial gating is an effective means for boosting information transfer during these tasks.

## Author contributions

AG, MK, and SS made substantial contributions to the conception and design of the study. AG facilitated the recruitment of participants, data collection, and analysis. AG drafted the article. MK and SS revised it critically for important intellectual content. All authors provided their final approval for this version to be published.

## Funding

NIH grant: 1 K25 NS061001 01A2.

### Conflict of interest statement

The authors declare that the research was conducted in the absence of any commercial or financial relationships that could be construed as a potential conflict of interest.
